# [Retracted] Tripartite motif 16 suppresses breast cancer stem cell properties through regulation of Gli‑1 degradation via the ubiquitin‑proteasome pathway

**DOI:** 10.3892/or.2024.8748

**Published:** 2024-05-13

**Authors:** Juntao Yao, Tao Xu, Tao Tian, Xiao Fu, Wenjuan Wang, Suoni Li, Tingting Shi, Aili Suo, Zhiping Ruan, Hui Guo, Yu Yao

Oncol Rep 35: 1204–1212, 2016; DOI: 10.3892/or.2015.4437

Following the publication of the above paper, it was drawn to the Editors' attention by a concerned reader that the data obtained from sphere-forming assay experiments shown in Figs. 4C-F and 8B and C, and western blotting data in Figs. 4A and 8A, were strikingly similar to data appearing in different form in other articles by different authors from different research institutes that had already been published, one of which has been retracted. Moreover, a pair of data panels comparing between Fig. 4E and 8C were partly overlapping, such that these data appear to have been derived from the same original source.

Owing to the fact that the contentious data in the above article had already been published elsewhere prior to its submission to *Oncology Reports*, the Editor has decided that this paper should be retracted from the Journal. The authors were asked for an explanation to account for these concerns, but the Editorial Office did not receive a reply. The Editor apologizes to the readership for any inconvenience caused.

